# Intra- and interobserver reproducibility of pancreatic perfusion by computed tomography

**DOI:** 10.1038/s41598-019-42519-w

**Published:** 2019-04-15

**Authors:** Tiago S. Garcia, Jean-Luc Engelholm, Michaël Vouche, Vânia N. Hirakata, Cristiane B. Leitão

**Affiliations:** 1 Postgraduate Program in Medical Sciences: Endocrinology, Universidade Federal do Rio Grande do Sul, Porto Alegre, RS Brazil; 2 Radiology Department, Institut Jules Bordet, Brussels, Belgium; 3 Grupo de Pesquisa e Pós-graduação do Hospital de Clínicas de Porto Alegre, Porto Alegre, RS Brazil; 4 Endocrine Division, Hospital de Clínicas de Porto Alegre, Porto Alegre, RS Brazil; 5 Radiology Department, Hospital de Clínicas de Porto Alegre, Porto Alegre, RS Brazil

## Abstract

The aim of this study was to measure intra- and interobserver agreement among radiologists in the assessment of pancreatic perfusion by computed tomography (CT). Thirty-nine perfusion CT scans were analyzed. The following parameters were measured by three readers: blood flow (BF), blood volume (BV), mean transit time (MTT) and time to peak (TTP). Statistical analysis was performed using the Bland-Altman method, linear mixed model analysis, and intraclass correlation coefficient (ICC). There was no significant intraobserver variability for the readers regarding BF, BV or TTP. There were session effects for BF in the pancreatic body and MTT in the pancreatic tail and whole pancreas. There were reader effects for BV in the pancreatic head, pancreatic body and whole pancreas. There were no effects for the interaction between session and reader for any perfusion parameter. ICCs showed substantial agreement for the interobserver measurements and moderate to substantial agreement for the intraobserver measurements, with the exception of MTT. In conclusion, satisfactory reproducibility of measurements was observed for TTP in all pancreatic regions, for BF in the head and BV in the tail, and these parameters seem to ensure a reasonable estimation of pancreatic perfusion.

## Introduction

Ultrasound, computed tomography (CT) and magnetic resonance imaging (MRI) can be used in tissue perfusion studies^1–7^. In CT, however, there is a linear relationship between the concentration of iodinated contrast media and the recorded density in Hounsfield units^8–10^, and this could be considered the preferred technique for the acquisition of perfusion images^11^. Perfusion CT is a relatively recent technique and can provide qualitative and quantitative information regarding tissue perfusion parameters in a non-invasive way. In comparing perfusion CT and dynamic contrast enhanced MRI, the major drawback of the first method is the use of ionizing radiation, while the second is a more complex and time-consuming method. However, CT is faster and more available than MRI. Furthermore, there are more restrictions in MRI use comparing to CT (e.g., implanted devices, metallic foreign bodies and prostheses). Variability of biomarkers in perfusion CT and dynamic contrast enhanced MRI are similar^12,13^.

In 1995, Miles *et al*.^14^ conducted the first study on the feasibility of pancreatic perfusion CT. Since then, a number of studies have used CT to observe normal pancreatic perfusion values, pancreatic perfusion impairments in pancreatic and hepatic diseases and modifications in pancreatic perfusion after oncologic therapy^8,11,15–27^.

However, the effects of observer variability in diagnostic testing can have a potentially large impact^28^. In clinical practice, radiologist-dependent factors may contribute to measurement inconsistencies due to variations in measurement technique or experience^29–34^. Therefore, intra- and interobserver variability must be assessed to guarantee the accuracy of radiologic readings.

Little information is available about intra- and interobserver variability in pancreatic perfusion CT^35,36^. Therefore, the purpose of this study is to measure intra- and interobserver agreement among radiologists with different levels of experience while assessing pancreatic perfusion by CT.

## Material and Methods

Between October 2015 and September 2016, we prospectively analyzed 12 scans from subjects who were referred for abdominal perfusion CT at the Jules Bordet Institute (Brussels, Belgium) for reasons unrelated to pancreatic symptoms or disease. Twenty-seven scans from a CT archive were also included. Informed consent was obtained from all participants that were prospectively included. The study was approved by the Ethics Committee of the Jules Bordet Institute and is in accordance with the Declaration of Helsinki. Exclusion criteria were pregnancy, history of allergic reaction to iodinated contrast media, renal insufficiency and history of pancreatic disease. Patients with altered pancreatic imaging (abnormal volume, morphology and/or composition, or focal lesions) were not excluded.

All patients were scanned in a Siemens Somatom^®^ Force 192-slice scanner (Munich, Germany). The perfusion CT examination was performed in the interval between unenhanced and portal phases. To define a correct delay for the perfusion CT, a test phase was performed after injecting 10 mL of nonionic contrast medium (Iomeron 400), followed by a 21 mL bolus of saline solution after an 8 s delay. For this test phase, a region of interest (ROI) was set on the distal thoracic aorta and 15 images were acquired (1 every 2 s, rotation time: 0.25 s, 40 mAs, 100Kvp), so that a curve of aortic enhancement could be obtained (software DynEva^®^, Siemens). The time required to achieve peak aortic enhancement was used to define the delay for the perfusion CT. Next, 50 mL of nonionic contrast medium (Iomeron 400) were injected through an 18-gauge catheter into an antecubital vein at a flow rate of 4.0 mL/s, followed by a 21 mL chaser bolus of saline solution. Eighty kilovolt peak (kVp) voltage was used for the CT tube. The dynamic imaging sequence consisted of 31 acquisitions of 0.25-second duration (rotation time) at an interval of 1.5 s (cycle time), resulting in a total examination time of 45.45 s. The perfusion sequence covered a craniocaudal width of 24 cm (collimation of 48 × 1.2 mm). A portal phase was acquired with a delay of 70 s after 70 mL of contrast media was injected intravenously at the end of the perfusion CT (Table 1).

**Table 1:** Protocol of CT acquisition.

CT Scanning parameters	Precontrast	Perfusion	Venous
Voltage (kVp)	90	80	90
Mean scanning delay after contrast injection (s)		test*	70
Collimation (mm)	192 × 0.6	48 × 1.2	128 × 0.6
Rotation time (s)	0.5	0.25 (full rotation) 1.5 (cycle time)	0.5
Pitch	0.6	0.6	0.8
Kernel	Br36	Br32	Br40
Slice thickness reconstructed (mm)	3	5	3
Contrast agent dose (mL)		50	70
Contrast injection rate (mL/s)		4	4
Number of acquisitions Bolus NaCl (mL)	Helical	31 21	helical 21

*Depends on test phase.

**Figure 1:**
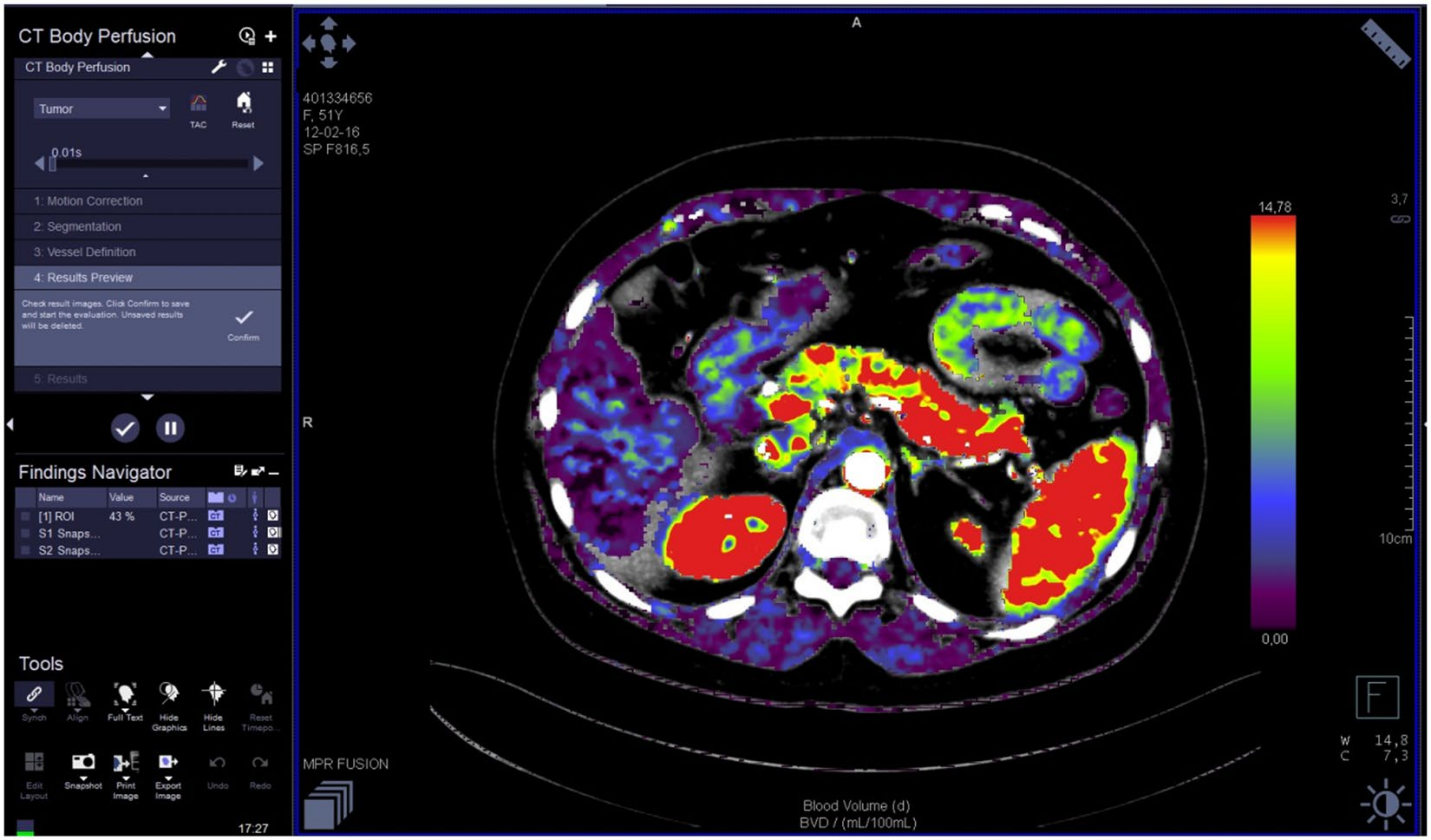
Perfusion CT: image processing.

**Figure 2:**
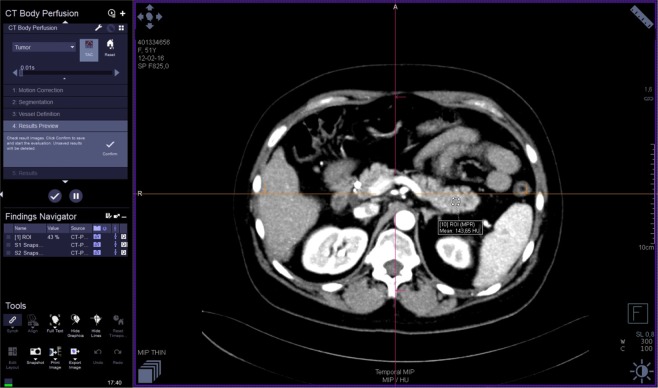
Perfusion CT: ROI positioning in pancreatic tail.

**Figure 3:**
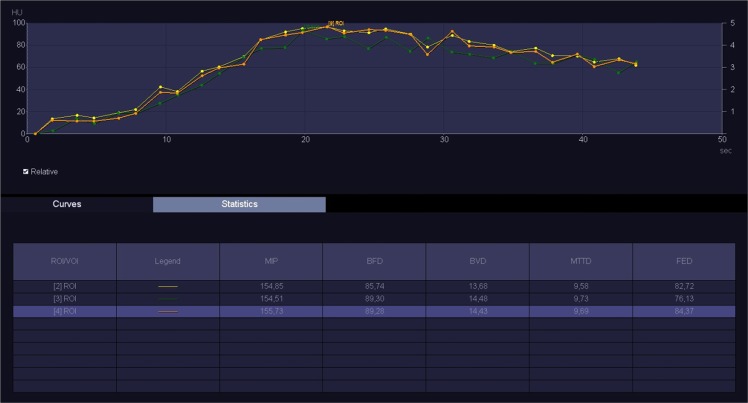
Perfusion CT: curves for blood flow, blood volume, mean transit time and time to peak.

The images were analyzed by three radiologists (J.L.E. – reader 1, T.S.G. - reader 2, M.V. – reader 3) with 25, 16 and six years of experience in abdominal imaging, respectively. Each reader performed two reading sessions with at least a 24 h interval. The image data were processed on a workstation (Syngo.via^®^, Siemens) with commercial perfusion CT software (CT Body Perfusion, Siemens) based on the maximum slope model. Motion correction was performed by using a commercial non-rigid motion correction algorithm (Syngo®.via Body Perfusion, Siemens) to improve anatomical alignment. The following parameters were measured: blood flow (measured in mL/100 mL/min), blood volume (measured in mL/100 mL), time to peak (measured in seconds), and mean transit time (measured in seconds). Arterial input was measured by automatically placed ROI in the abdominal aorta. To obtain perfusion CT parameters, each radiologist manually drew three non-superposable circular ROI (between 1.0 and 2.0 cm²) in the head, three in the body and three in the tail of the pancreas to measure these parameters, avoiding visible vessels and ducts. The mean ROI value for each parameter in each part of the pancreas was considered for analysis. The parameters for the whole pancreas were calculated as the sum of the values of the pancreatic head, body and tail divided by three. An example of perfusion CT image processing is shown in Figs 1–3.

### Statistical analysis

The statistical packages utilized were R-project and SPSS (version 18). Mean and standard deviation were used to describe the analyzed variables. Intraobserver agreement was evaluated by the Bland-Altman method and Student’s *t*-test for paired samples. Linear mixed model analysis was performed to determine interobserver reliability, considering session and reader effects. The significance level was set at 0.05. Intraclass correlation coefficients ICCs were also calculated to analyze both intra- and interobserver agreement, interpreted by using the following categories: 0.00–0.10 = virtually none; 0.11–0.40 = slight; 0.41–0.60 = fair; 0.61–0.80 = moderate and 0.81–1.00 = substantial agreement^37^.

### Approval, accordance and informed consent statement

The study was approved by the Ethics Committee of the Jules Bordet Institute (Brussels, Belgium) and is in accordance with the Declaration of Helsinki. Informed consent was obtained from all participants that were prospectively included.

## Results

A total of 39 patients [men: n = 21 (53.8%)] were included, with a mean age of 64 years. Seventeen patients had type 2 diabetes mellitus (DM2) and 22 did not. Two patients were excluded due to technical problems, which lead to difficulties in measuring pancreatic perfusion parameters (large ascites and improper contrast media injection).

**Figure 4:**
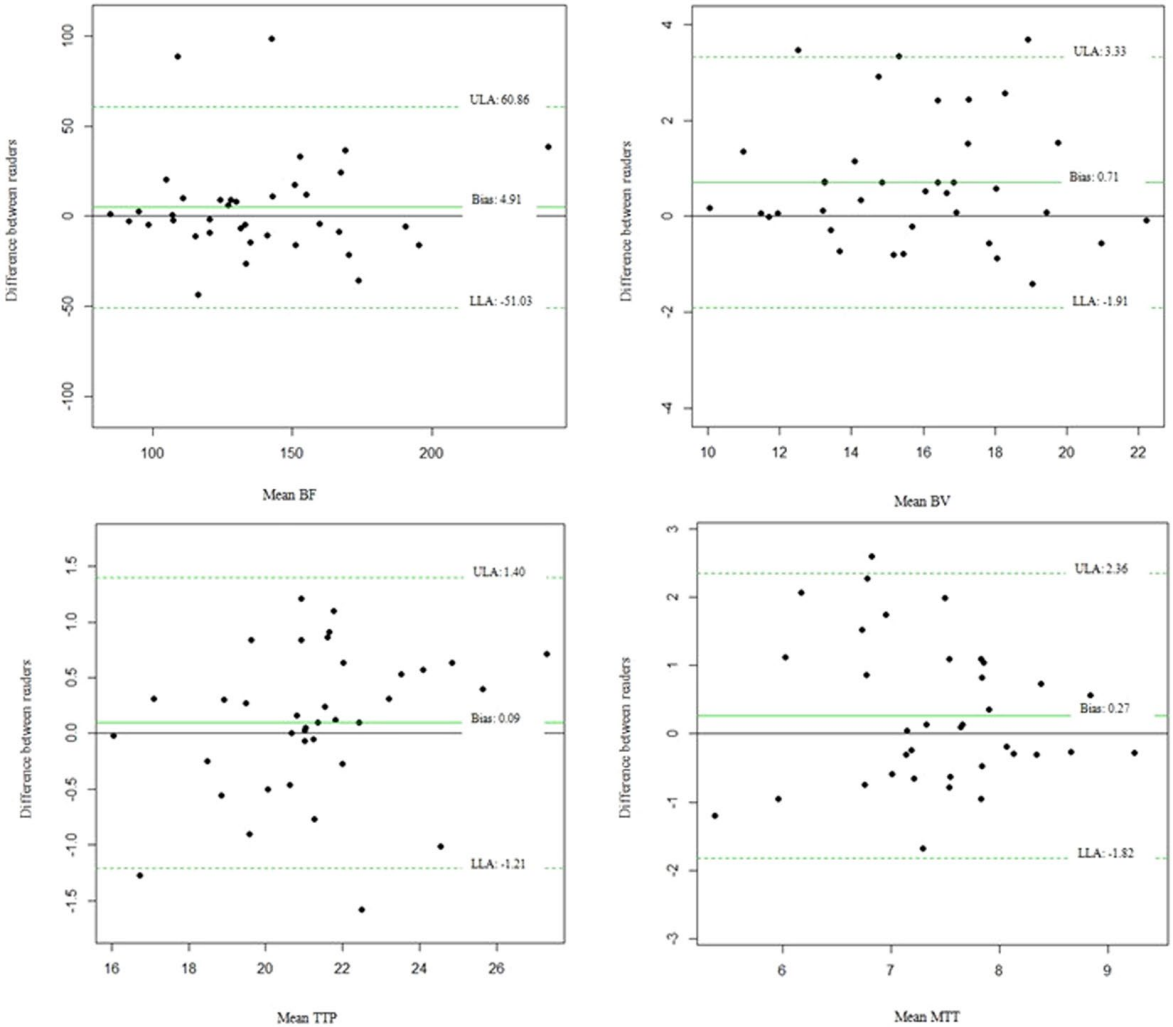
Bland-Altman plots graphics showing interobserver variability between readers 1 and 2 in blood flow (BF, mL/100 mL/min), blood volume (BV, mL/100 mL), time to peak (TTP, seconds) and mean transit time (MTT, seconds); ULA: upper limit of agreement; LLA: lower limit of agreement.

The Bland-Altman analysis showed no significant intraobserver variability for readers 1 or 2 regarding BF, BV, MTT and TTP; for reader 3, there was significant variability for BF in the pancreatic tail and the whole pancreas (tail - mean difference: 11.49 mL/100 mL/min ± 27.6, 95% limit of agreement: −42.6 ± 65.5, p = 0.016; whole pancreas - mean difference: 6.04 mL/100 mL/min ± 13.0, 95% limit of agreement: −19.4 ± 31.5, p = 0.008) and for MTT in the pancreatic tail and whole pancreas (tail - mean difference: −0.47 s ± 1.2, 95% limit of agreement: −2.92 ± 2.0, p = 0.027; whole pancreas - mean difference: −0.26 s ± 0.8, 95% limit of agreement: −1.7 ± 1.2, p = 0.048). The ICCs showed an overall moderate to substantial agreement for the intraobserver measurements, with the exception of MTT in all pancreatic regions (Table 2). Bland-Altman plots graphics with interobserver variability between readers 1 and 2 in BF, BV, TTP and MTT in the whole pancreas are shown in Fig. 4. Table 3 summarizes the pancreatic perfusion measurements for each session and reader, as well as their respective effects and the interaction effect between session and reader. There were session effects on BF in the pancreatic body (mean difference: 4.5 mL/100 mL/min ± 2.20, p = 0.048) and on MTT in the pancreatic tail (mean difference: 0.28 s ± 0.11, p = 0.021) and the whole pancreas (mean difference: 0.18 s ± 0.06, p = 0.007). There were reader effects on BV in pancreatic head, pancreatic body and the whole pancreas. No session effects were found on BV or TTP and no reader effects were found on BF, MTT or TTP. There were no interaction effects between session and reader for any perfusion parameter. ICCs for the interobserver measurements on pancreatic perfusion CT parameters showed an overall substantial agreement, with the exception of MTT in the body, tail and whole pancreas, where it was only fair (Table 4).

**Table 2:** Intraobserver variability. Bland-Altman analysis (mean difference) and ICCs for pancreatic perfusion parameters.

	Reader 1			Reader 2			Reader 3		
	(n = 37)			(n = 37)			(n = 37)		
	Mean difference (95% Limit of agreement)	ICC (95% Confidence interval)	p	Mean difference (95% Limit of agreement)	ICC (95% Confidence interval)	p	Mean difference (95% Limit of agreement)	ICC (95% Confidence interval)	p
BF head	−1.78 (−47.9;44.3)	0.81 (0.66;0.90)	0.657	−2.81 (−50.4;44.8)	0.80 (0.65;0.89)	0.486	3.76 (−29.4;36.9)	0.88 (0.79;0.94)	0.185
BF body	4.42 (−33.2;42.0)	0.88 (0.77;0.93)	0.182	6.37 (−36.2;48.9)	0.84 (0.72;0.92)	0.083	2.87 (34.8;40.5)	0.86 (0.75;0.93)	0.369
BF tail	0.04 (−42.3;42.4)	0.83 (0.69;0.91)	0.992	4.80 (−86.7;96.3)	0.52 (0.24;0.72)	0.535	11.49 (−42.6;65.5)	0.68 (0.45;0.83)	0.016
BF whole pancreas	0.89 (−32.4;34.2)	0.89 (0.79;0.94)	0.757	2.79 (−43.3;48.8)	0.81 (0.66;0.90)	0.475	6.04 (−19.4;31.5)	0.91 (0.81;0.96)	0.008
BV head	−0.01 (−3.5;3.5)	0.85 (0.72;0.92)	0.425	−0.14 (−3.9;3.6)	0.85 (0.73;0.92)	0.657	−0.09 (−3.8;3.6)	0.86 (0.75;0.93)	0.768
BV body	−0.23 (−4.0;3.6)	0.86 (0.75;0.93)	0.475	0.22 (−3.1;3.5)	0.87 (0.77;0.93)	0.425	0.00 (−3.2;3.2)	0.90 (0.82;0.95)	1
BV tail	0.24 (−4.3;4.3)	0.80 (0.64;0.89)	0.947	0.22 (4.0;4.4)	0.81 (0.66;0.90)	0.546	−0.37 (−4.0;3.2)	0.82 (0.68;0.90)	0.227
BV whole pancreas	0.09 (−2.8;2.6)	0.90 (0.81;0.95)	0.701	0.10 (−2.0;2.9)	0.94 (0.88;0.97)	0.567	−0.15 (−2.5;2.2)	0.93 (0.86;0.96)	0.441
MTT head	−0.003 (−2.3;2.3)	0.62 (0.37;0.78)	0.985	0.05 (−2.6;2.7)	0.54 (0.26;0.73)	0.808	−0.17 (−2.4;2.1)	0.58 (0.32;0.76)	0.365
MTT body	−0.18 (−2.5;2.2)	0.70 (0.49;0.83)	0.366	−0.28 (−2.2;1.6)	0.67 (0.45;0.82)	0.093	−0.12 (−2.3;2.0)	0.58 (0.31;0.76)	0.517
MTT tail	−0.16 (−2.3;2.0)	0.65 (0.42;0.80)	0.375	−0.19 (−3.0;2.6)	0.58 (0.32;0.76)	0.415	−0.47 (−2.92;2.0)	0.33 (0.03;0.58)	0.027
MTT whole pancreas	−0.11 (−1.9;1.7)	0.69 (0.47;0.83)	0.450	−0.17 (−2.0;1.6)	0.62 (0.38;0.79)	0.267	−0.26 (−1.7;1.2)	0.60 (0.34;0.77)	0.048
TTP head	0.08 (−3.4;3.6)	0.78 (0.61;0.88)	0.785	−0.28 (−2.8;2.7)	0.86 (0.74;0.93)	0.904	−0.03 (−3.4;3.3)	0.77 (0.59;0.87)	0.923
TTP body	−0.04 (−2.8;2,7)	0.83 (0.69;0.91)	0.864	−0.23 (−3.5;3.1)	0.83 (0.69;0.91)	0.407	−0.28 (−4.7;4.2)	0.64 (0.40;0.80)	0.464
TTP tail	0.47 (−2.6;3.5)	0.78 (0.62;0.88)	0.079	0.08 (−2.9;3.1)	0.83 (0.69;0.91)	0.744	−0.17 (−2.9;2.5)	0.81 (0.66;0.90)	0.467
TTP whole pancreas	0.17 (−1.6;2.0)	0.92 (0.85;0.96)	0.269	−0.06 (−1.7;1.6)	0.94 (0.89;0.97)	0.671	−0.16 (−2.1;1.8)	0.89 (0.80;0.94)	0.352

BF: blood flow in mL/100 mL/min; BV: blood volume in mL/100 mL; MTT: mean transit time in s; TTP: time to peak in s; ICC: intraclass correlation coefficient.

**Table 3:** Pancreatic perfusion measurements and effects of session, reader, and interaction between session and reader.

	Reader 1		Reader 2		Reader 3		Effect of session	Effect of reader	Effect of interaction session*reader
	Session 1	Session 2	Session 1	Session 2	Session 1	Session 2			
BF head	132.87 ± 33.9	135.14 ± 39.6	130.94 ± 35.8	133.76 ± 41.0	134.19 ± 36.3	130.43 ± 34.6	0.817	0.621	0.310
BF body	142.42 ± 39.2	138.13 ± 37.2	139.90 ± 36.0	133.53 ± 43.3	137.12 ± 39.2	134.25 ± 34.0	0.048	0.171	0.732
BF tail	144.24 ± 32.7	144.84 ± 38.1	153.57 ± 42.0	148.76 ± 52.5	149.01 ± 40.7	137.51 ± 31.5	0.073	0.143	0.074
BF whole pancreas	139.85 ± 32.8	139.37 ± 35.7	141.47 ± 33.2	138.68 ± 42.1	140.11 ± 35.9	134.06 ± 31.3	0.087	0.086	0.304
BV head	15.21 ± 3.3	15.22 ± 3.1	15.77 ± 3.5	15.91 ± 3.6	15.76 ± 3.6	15.86 ± 3.5	0.589	0.027	0.966
BV body	15.01 ± 3.3	15.24 ± 4.0	16.06 ± 3.4	15.84 ± 3.2	15.40 ± 3.7	15.40 ± 3.6	0.985	0.045	0.633
BV tail	15.72 ± 3.2	15.74 ± 3.5	16.51 ± 3.3	16.29 ± 3.6	15.90 ± 3.0	16.27 ± 3.1	0.776	0.142	0.484
BV whole pancreas	15.32 ± 3.0	15.40 ± 3.1	16.12 ± 2.9	16.02 ± 3.1	15.69 ± 3.1	15.84 ± 3.1	0.591	0.009	0.637
MTT head	7.51 ± 1.4	7.52 ± 1.3	7.84 ± 1.3	7.79 ± 1.4	7.71 ± 1.3	7.89 ± 1.2	0.623	0.075	0.673
MTT body	7.15 ± 1.5	7.33 ± 1.6	7.53 ± 1.1	7.82 ± 1.3	7.36 ± 1.2	7.48 ± 1.1	0.093	0.137	0.741
MTT tail	7.14 ± 1.3	7.30 ± 1.3	7.25 ± 1.6	7.44 ± 1.3	7.19 ± 1.3	7.67 ± 0.8	0.021	0.490	0.515
MTT whole pancreas	7.27 ± 1.2	7.38 ± 1.1	7.51 ± 1.0	7.68 ± 1.1	7.42 ± 1.0	7.68 ± 0.8	0.007	0.222	0.819
TTP head	21.72 ± 2.7	21.64 ± 2.6	21.54 ± 2.8	21.56 ± 2.5	21.48 ± 2.7	21.51 ± 2.2	0.964	0.680	0.932
TTP body	21.01 ± 2.3	21.05 ± 2.4	21.12 ± 2.9	21.35 ± 2.7	21.07 ± 2.6	21.35 ± 2.7	0.350	0.364	0.834
TTP tail	21.14 ± 2.7	20.67 ± 2.2	21.16 ± 2.5	21.08 ± 2.6	20.76 ± 2.2	20.93 ± 2.2	0.370	0.431	0.218
TTP whole pancreas	21.29 ± 2.4	21.12 ± 2.2	21.27 ± 2.5	21.33 ± 2.4	21.10 ± 2.3	21.26 ± 2.0	0.877	0.653	0.316

BF: blood flow in mL/100 mL/min; BV: blood volume in mL/100 mL; MTT: mean transit time in s; TTP: time to peak in s.

**Table 4:** Interobserver ICCs and CI 95% for pancreatic perfusion parameters.

	Reader 1 × 2	Reader 1 × 3	Reader 2 × 3
BF head	0.94 (0.88;0.97)	0.98 (0.95;0.99)	0.97 (0.93;0.98)
BF body	0.91 (0.83;0.95)	0.96 (0.91;0.98)	0.94 (0.89;0.97)
BF tail	0.79 (0.59;0.89)	0.92 (0.84;0.96)	0.88 (0.75;0.94)
BF whole pancreas	0.93 (0.86;0.96)	0.98 (0.95;0.99)	0.96 (0.92;0.98)
BV head	0.94 (0.86;0.97)	0.95 (0.88;0.97)	0.97 (0.94;0.99)
BV body	0.88 (0.76;0.94)	0.92 (0.85;0.96)	0.93 (0.86;0.97)
BV tail	0.89 (0.78;0.94)	0.89 (0.79;0.94)	0.90 (0.81;0.95)
BV whole pancreas	0.94 (0.84;0.97)	0.96 (0.91;0.98)	0.97 (0.94;0.98)
MTT head	0.83 (0.67;0.91)	0.87 (0.74;0.94)	0.92 (0.85;0.96)
MTT body	0.56 (0.17;0.77)	0.68 (0.37;0.83)	0.75 (0.51;0.87)
MTT tail	0.56 (0.13;0.77)	0.64 (0.30;0.81)	0.60 (0.23;0.80)
MTT whole pancreas	0.63 (0.30;0.81)	0.77 (0.55;0.88)	0.82 (0.65;0.91)
TTP head	0.94 (0.88;0.97)	0.91 (0.82;0.95)	0.90 (0.80;0.95)
TTP body	0.96 (0.92;0.98)	0.87 (0.74;0.93)	0.83 (0.67;0.91)
TTP tail	0.94 (0.89;0.97)	0.90 (0.80;0.95)	0.88 (0.77;0.94)
TTP whole pancreas	0.98 (0.96;0.99)	0.94 (0.88;0.97)	0.93 (0.87;0.97)

BF: blood flow in mL/100 mL/min; BV: blood volume in mL/100 mL; MTT: mean transit time in s;

TTP: time to peak in s; ICC: intraclass correlation coefficient; CI: confidence interval.

## Discussion

Our results showed good overall intraobserver agreement for pancreatic perfusion CT parameters, except for BF in the pancreatic tail for reader 3, who had the least experience, and for MTT in all regions of the pancreas for the three readers. No session effects were found on BV or TTP, and there were no reader effects on BF or MTT. TTP values were not significantly different between readers or reading sessions. However, there were session effects on BF in the pancreatic body and on MTT in the pancreatic tail and the whole pancreas. This is probably because the measurements in the body and the tail of the pancreas are more difficult to obtain due to the smaller thickness and the greater variation of the morphology of the pancreas in these regions. Thus, some variability may occur between different sessions of the same reader. Reader effects were found on BV in the pancreatic head and body and the whole pancreas. The measurements obtained by our less experienced (reader 3), seemed different than those obtained by our two most experienced readers. Possibly this is because no training sessions have been held prior to the measurements, suggesting that training sessions for inexperienced readers should be performed. Of note, BF in the head, BV in the tail, and TTP in all pancreatic regions showed good intraobserver correlation and no significant reader or session effects, which supports the use of these parameters in pancreatic perfusion CT.

Measurement reproducibility and accuracy are of particular interest in radiology, since important clinical decisions are often based on CT measurements^38,39^. Accordingly to McErlean *et al*.^40^, “lesion measurements on images should be accurate, reproducible, and performed in a standardized fashion with low rates of intra- and interobserver variability”. Li *et al*.^35^ reported an interobserver correlation over 0.9 for BF and BV in normal pancreas. Xie *et al*.^36^ also observed substantial agreement (0.85). Our study evaluated intraobserver agreement by two methods: Bland-Altman and mixed model analysis; interobserver correlation was evaluated by mixed model analysis, considering session and reader effects. It is important to emphasize that we obtained perfusion measurements in pancreas without lesions, which may not reflect clinical practice where perfusion CT may be used to assess focal pancreatic lesions. This issue could be addressed in forthcoming studies”.

This study has some limitations. Our results are based on the readings of only three radiologists. This small number of observers may not truly represent the community of radiologists. The measures obtained by our least experienced reader (reader 3) showed poor intraobserver agreement for BV and MTT in the pancreatic tail and the whole pancreas in the Bland-Altman analysis, suggesting that a preliminary training session, which was not performed in this study, could improve intraobserver results. However, no differences attributable to a single reader were found in mixed model analysis. Second, we only applied maximal slope model to calculate perfusion parameters. There are many other methods that can be used for this purpose, including deconvolution model and dual-input single compartment, and these algorithms are not interchangeable. Although there is no consensus about the best method, maximal slope model is used in the majority of the studies on CT perfusion. Third, some reading sessions of the same patient were performed by a reader with a 24 hour interval. In such a short interval, the intraobserver results can be affected by an observer’s recognition of an image (memory bias).

In conclusion, our data support the use of pancreatic perfusion CT by radiologists of different levels of experience. BF in the head, BV in the tail, and TTP in all pancreatic regions seem to be the best parameters to ensure a reasonably reliable reproducibility for pancreatic perfusion CT.

## Data Availability

All data generated or analyzed during this study are included in this published article.

## References

[1] Doi R (1988). Simultaneous measurement of hepatic arterial and portal venous flows by transit time ultrasonic volume flowmetry. Surgery, Gynecology & Obstetrics..

[2] Kleber G (1999). Hepatic arterial flow volume and reserve in patients with cirrhosis: use of intra-arterial Doppler and adenosine infusion. Gastroenterology..

[3] Blomley MJ (1995). Liver perfusion studied with ultrafast CT. Journal of computer assisted tomography..

[4] Materne R (2002). Assessment of hepatic perfusion parameters with dynamic MRI. Magnetic Resonance in Medicine..

[5] Hirshberg B (2009). Pancreatic perfusion of healthy individuals and type 1 diabetic patients as assessed by magnetic resonance perfusion imaging. Diabetologia..

[6] Bali MA (2008). Pancreatic perfusion: noninvasive quantitative assessment with dynamic contrast-enhanced MR imaging without and with secretin stimulation in healthy volunteers–initial results. Radiology..

[7] Schraml C, Schwenzer NF, Martirosian P, Claussen CD, Schick F (2008). Perfusion imaging of the pancreas using an arterial spin labeling technique. Journal of Magnetic Resonance Imaging.

[8] Park MS (2009). Perfusion CT: noninvasive surrogate marker for stratification of pancreatic cancer response to concurrent chemo- and radiation therapy. Radiology..

[9] Miles K A (2003). Perfusion CT for the assessment of tumour vascularity: which protocol?. The British Journal of Radiology.

[10] Kambadakone AR, Sharma A, Catalano OA, Hahn PF, Sahani DV (2011). Protocol modifications for CT perfusion (CTp) examinations of abdomen-pelvic tumors: impact on radiation dose and data processing time. European Radiology..

[11] Kandel S (2009). Whole-organ perfusion of the pancreas using dynamic volume CT in patients with primary pancreas carcinoma: acquisition technique, post-processing and initial results. European Radiology..

[12] Ng CS (2010). Reproducibility of Perfusion Parameters in Dynamic Contrast-Enhanced MRI of Lung and Liver Tumors: Effect on Estimates of Patient Sample Size in Clinical Trials and on Individual Patient Responses. American Journal of Roentgenology..

[13] Campbell BC (2012). Comparison of Computed Tomography Perfusion and Magnetic Resonance Imaging Perfusion-Diffusion Mismatch in Ischemic Stroke. Stroke..

[14] Miles KA, Hayball MP, Dixon AK (1995). Measurement of human pancreatic perfusion using dynamic computed tomography with perfusion imaging. The British Journal of Radiology..

[15] Cui B, Zhao C, He J, Zhang X (2011). Whole-organ CT perfusion imaging of the pancreas in patients with type 2 Diabetes. Chinese Journal of Medical Imaging Technology..

[16] Bize PE, Platon A, Becker CD, Poletti PA (2006). Perfusion measurement in acute pancreatitis using dynamic perfusion MDCT. American Journal of Roentgenology..

[17] D’Onofrio M (2013). Perfusion CT can predict tumoral grading of pancreatic adenocarcinoma. European Journal of Radiology..

[18] Delrue L (2012). Tissue perfusion in pathologies of the pancreas: assessment using 128-slice computed tomography. Abdominal imaging..

[19] Klauss M (2012). Computed tomography perfusion analysis of pancreatic carcinoma. Journal of Computer Assisted Tomography..

[20] Klauss M (2013). l. Dual-energy perfusion-CT of pancreatic adenocarcinoma. European Journal of Radiology..

[21] Tsushima Y, Kusano S (1998). Age-dependent decline in parenchymal perfusion in the normal human pancreas: measurement by dynamic computed tomography. Pancreas..

[22] Abe H (2005). Quantitative tissue blood flow evaluation of pancreatic tumor: comparison between xenon CT technique and perfusion CT technique based on deconvolution analysis. Radiation Medicine..

[23] d’Assignies G (2009). Pancreatic endocrine tumors: tumor blood flow assessed with perfusion CT reflects angiogenesis and correlates with prognostic factors. Radiology..

[24] Watanabe T (2011). Relationship between serum angiopoietin-2 level and perfusion CT parameters in severe acute pancreatitis. The American Journal of Gastroenterology..

[25] Kanda T (2012). Perfusion measurement of the whole upper abdomen of patients with and without liver diseases: initial experience with 320-detector row CT. European Journal of Radiology..

[26] Motosugi U (2012). Multi-organ perfusion CT in the abdomen using a 320-detector row CT scanner: preliminary results of perfusion changes in the liver, spleen, and pancreas of cirrhotic patients. European Journal of Radiology..

[27] Yao JC (2015). Perfusion computed tomography as functional biomarker in randomized run-in study of bevacizumab and everolimus in well-differentiated neuroendocrine tumors. Pancreas..

[28] Mower WR (1999). Evaluating bias and variability in diagnostic test reports. Annals of Emergency Medicine..

[29] Lederle FA (1995). Variability in measurement of abdominal aortic aneurysms. Abdominal Aortic Aneurysm Detection and Management Veterans Administration Cooperative Study Group. Journal of Vascular Surgery..

[30] Oxnard GR (2011). Variability of lung tumor measurements on repeat computed tomography scans taken within 15 minutes. Journal of Clinical Oncology..

[31] Thiesse P (1997). Response rate accuracy in oncology trials: reasons for interobserver variability. Groupe Francais d’Immunotherapie of the Federation Nationale des Centres de Lutte Contre le Cancer. Journal of Clinical Oncology..

[32] Wormanns D, Diederich S, Lentschig MG, Winter F, Heindel W (2000). Spiral CT of pulmonary nodules: interobserver variation in assessment of lesion size. European Radiology..

[33] Zhao B (2005). Pulmonary metastases: effect of CT section thickness on measurement-initial experience. Radiology..

[34] Bankier AA, Levine D, Halpern EF, Kressel HY (2010). Consensus interpretation in imaging research: is there a better way?. Radiology..

[35] Li HO (2014). Low-dose whole organ CT perfusion of the pancreas: preliminary study. Abdominal Imaging..

[36] Xie Q (2013). Whole-organ CT perfusion of the pancreas: impact of iterative reconstruction on image quality, perfusion parameters and radiation dose in 256-slice CT-preliminary findings. PloS One..

[37] Bretas EAS (2017). Is liver perfusion CT reproducible? A study on intra and interobserver agreement of normal hepatic haemodynamic parameters obtained with two different software packages. British Journal of Radiology..

[38] Edge SB, Compton CC (2010). The American Joint Committee on Cancer: the 7th edition of the AJCC cancer staging manual and the future of TNM. Annals of Surgical Oncology..

[39] Eisenhauer EA (2009). New response evaluation criteria in solid tumours: revised RECIST guideline (version 1.1). Eur J Cancer..

[40] McErlean A (2013). Intra- and interobserver variability in CT measurements in oncology. Radiology..

